# Systematic review of experiences and perceptions of key actors and organisations at multiple levels within health systems internationally in responding to COVID-19

**DOI:** 10.1186/s13012-021-01114-2

**Published:** 2021-05-07

**Authors:** Simon Turner, Natalia Botero-Tovar, Maria Alejandra Herrera, Juan Pablo Borda Kuhlmann, Francisco Ortiz, Jean Carlo Ramírez, Luisa Fernanda Maldonado

**Affiliations:** School of Management, University of los Andes, Bogotá, Colombia

**Keywords:** COVID-19, Healthcare workers, Health administration, Qualitative, Systematic review

## Abstract

**Background:**

COVID-19 has presented challenges to healthcare systems and healthcare professionals internationally. After one year of the pandemic, the initial evidence on health system responses begins to consolidate, and there is a need to identify and synthesise experiences of responding to COVID-19 among healthcare professionals and other health system stakeholders. This systematic review of primary qualitative studies depicts the experiences and perceptions of organisations and actors at multiple levels of health systems internationally in responding to COVID-19.

**Methods:**

Six main databases of biomedical information, public health and health administration research were searched over the period October 1, 2019, to October 21, 2020. Information extracted from included studies was analysed thematically.

**Results:**

Thirty-four studies were eligible for data extraction. Nine of those studies, of lower methodological quality, were removed from the thematic analysis of study results. Considering the professional level experiences, predominant themes of the studies consisted of the new roles and responsibilities of healthcare workers, burnout and distress, recognition of ´unseen´ healthcare workers, and positive changes and emergent solutions amid the crisis. Organisational level findings of the studies included provision of psychological support, COVID-19 as "catalyst" for change, and exercise of more "open" leadership by managers and health authorities. Continuous training, regulation of working conditions, providing supportive resources, coordinating a diversity of actors, and reviewing and updating regulations were roles identified  at the local health system level.

**Conclusions:**

The experiences of frontline healthcare workers have been the focus of attention of the majority of primary qualitative studies as of October 2020. However, organisational and wider system level studies indicate that some responses to COVID-19 have been characterised by increased emphasis on coordination activities by local health system actors, making service adaptations at pace, and reliance on expanded roles of front-line workers. The need for theory-informed qualitative studies was identified at the organisational level.

**Trial registration:**

CRD42020202875

**Supplementary Information:**

The online version contains supplementary material available at 10.1186/s13012-021-01114-2.

Contributions to the literature

- This systematic review of primary qualitative studies collected multi-level responses (professional, organisational, local health system) to COVID-19 internationally.
- For implementation scientists, this review provides evidence of the necessity of system-wide interventions that safeguard the working conditions, training and physical and mental health of front-line healthcare workers.
- Analysis of organisational and wider system level processes indicate that some responses to COVID-19 have been characterised by emphasis on local health system level coordination, making service adaptations at pace and reliance on expanded roles of front-line workers.

## Background

COVID-19 is posing a major and unprecedented challenge to health service planning and delivery across health systems internationally [1]. Despite the uncertainty lived in the early months of the pandemic, evidence worldwide is consolidating; its analysis through systematic review creates an opportunity for implementation science to inform actions at later phases of the pandemic [2]. The experiences of, and burden placed on, healthcare workers and other actors in responding to the pandemic has been highlighted as an urgent need for discussion in the international literature [3]. A call for actions at different levels of health systems has been made, especially towards hospital managers and other leaders, to identify ways of mitigating the fear and distress among the healthcare workforce involved in responding to COVID-19 over a sustained period [4]. However, there is a lack of systematic research on experiences of COVID-19 at different levels of health systems, including lessons from professional, organisational and local system responses, that can be used to inform managerial and policy responses.

The aim of this systematic review of primary qualitative studies was to collect multi-level (professional, organisational, local health system) responses of health systems to COVID-19 internationally, with particular emphasis on the experiences of the organisations and actors providing health services who have been directly involved in, or affected by, the pandemic. Researchers worldwide have published regarding the experiences of healthcare providers, the capacity and adaptability of health systems, and the challenges presented to health systems by this pandemic [5]. This systematic review contributes to this literature by identifying and summarising experiences and perceptions of organisations and actors at multiple levels of health systems internationally in responding to COVID-19.

The question addressed by this review is: what are the experiences and perceptions of organisations and actors at multiple levels of health systems internationally in responding to COVID-19? This question was chosen because COVID-19 has posed an unprecedented threat to health systems [1], and it is important to learn from how actors and organisations at different levels of health systems internationally have experienced this threat and responded. For implementation science, the review examines how responses to COVID-19 have been negotiated and implemented in a context of ‘crisis’ which deviates from ‘usual care’ planning and its improvement [2]. Other systematic reviews have assessed risk factors and the development of the disease [6, 7] and treatments developed worldwide [8, 9].

This systematic review differs from other recently published reviews by synthesising contributions from primary qualitative studies at three levels: the experiences of healthcare professionals (both front-line and managerial staff); planning and provider organisations’ responses; and the coordinating roles of actors at the local health system level (e.g. metropolitan area- or region-spanning bodies such as professional associations, educational institutions, and funding and regulatory agencies), in relation to the COVID-19 pandemic in 2020.

## Methods

This systematic review is reported using the Preferred Reporting Items for Systematic Reviews and Meta-Analyses (PRISMA) guidelines (Additional file 1); the protocol is published in PROSPERO (see https://www.crd.york.ac.uk/prospero/display_record.php?ID=CRD42020202875).

### Information sources and search strategies

The studies were identified and chosen using six medical and management databases searched on October 21, 2020: PubMed, EBSCO Business Source Complete, OVID, APA PsycNet, Web of Science, and Scopus. A specific search strategy was designed for each one. The initial search formula was built for PubMed, which combined MeSH terms and keywords, and was later adapted to other databases depending on their format. Google Scholar was searched during scoping research prior to the formal search of bibliographical databases. Full search formulas are depicted in Additional file 2. A research librarian assisted with developing the search strategies.

### Study selection

Eligible studies needed to employ qualitative methods and analyse health system responses to the COVID-19 pandemic. The review focussed on qualitative studies because we were interested in ascertaining experiences and perceptions of the pandemic among key actors and organisations within health systems internationally. Inclusion criteria: (a) article reporting a primary qualitative study addressing COVID-19 pandemic; (b) date of publication after October 1, 2019; (c) the study is related to health systems, or healthcare organisations, or healthcare management, or healthcare professionals, or healthcare innovation, or healthcare improvement, or healthcare reform; (d) the study involves frontline healthcare workers or staff organisational decision-makers, or government policy makers as participants; and (e) mixed methods studies where qualitative data collection and analysis was included.

Exclusion criteria: (a) date of publication before October 2019; (b) articles related with patient outcomes or experiences that do not consider the organisational context of care; (c) studies published in languages other than English or Spanish (while translation software could have been used to translate articles not published in these languages, the authors were not confident of detecting linguistic nuances when reviewing studies in other languages); (d) studies that do not use qualitative research methods; (e) clinical studies about the diagnosis or treatment of COVID 19; (f) study protocols; (g) pre-prints or unpublished articles; (h) short communications, letters, editorials or commentaries that do not present primary qualitative data; and (i) systematic reviews. Grey or non-academic literature was excluded because we wanted to base any policy implications on robustly designed qualitative studies. Two protocols were excluded: one for a research project led by one of the authors [10]; the other for a mixed methods study of professional level responses to COVID-19 [11]. Neither had published results at the time of the searches.

### Assessment of eligibility

There were two phases in which all references were screened in accordance with a PRISMA flow diagram [12]. During the first phase, all titles/abstracts of references generated through the databases searches were screened for eligibility by six researchers. Following title/abstract screening, the full text of potentially relevant references was reviewed against the inclusion criteria. Articles that did not fulfil the criteria during either title/abstract or full text review were excluded and classified according to exclusion criteria. Eligibility of references was assessed by at least two researchers independently. In case of disagreement or doubt about eligibility, discussion among our research team was used to address debate over inclusion or exclusion.

### Quality assessment

Quality assessment criteria were applied during data extraction from all eligible articles that met the inclusion criteria. Quality assessment was performed initially by different researchers, then reviewed for consistency by one researcher (ST). The quality assessment tool was derived from Hawker et al. [13] (Additional file 3). The importance of applying this tool stems from the desire to include robust studies from which implications can be drawn to inform policy and practice. The process of conducting the review indicated that the pandemic had attracted a wealth of literature, including qualitative studies, of varying methodological quality. To assess study quality, the tool provides nine questions which can be answered ‘good,’ ‘fair,’ ‘poor’ or ‘very poor’ which are then, in turn, converted into a numerical score by assigning the answers from 1 point (very poor) to 4 points (good). The total score gave each qualitative study an A, B or C grade. While data were extracted and summarised from C grade articles, these studies were removed from the thematic analysis. This approach to reporting lower quality studies, and their removal from the analysis, has been used in previous reviews [14, 15].

### Data extraction and analysis of the results

These characteristics were identified and extracted in each study: type of qualitative study, sample size and characteristics, country, and main findings. The findings contained in the studies reviewed were analysed thematically using deductive and inductive approaches [16]. A multi-level approach to context was used deductively [14], whereby actors’ and organisations’ experiences of COVID-19 were divided into contextual levels: professional, organisational and local system. We focussed on data that relates to contextual levels because these are known to shape how health care phenomena are perceived [17] and influence the adaptation and spread of innovations [18, 19]. This deductive focus oriented data analysis towards how the phenomena of COVID-19 were experienced and responses made at different contextual levels of health systems.

Within each category, extracted data were analysed inductively in order to develop subcategories that characterised the responses at each level. This information was extracted into an Excel document paying attention to experiences and responses at the professional, organisational and local system levels. The professional level summarised the experiences and perspectives of the staff of the frontline; the organisational level depicted the strategies, key tasks, structure and organisational culture, including details on human resource management, where applicable; and the local system level (e.g. a metropolitan area or region) focussed on relationships between organisations (e.g. relationships between providers, between planners and providers, and the roles of other coordinating actors within local health systems).

## Results

### Identification of the studies

**Fig. 1:**
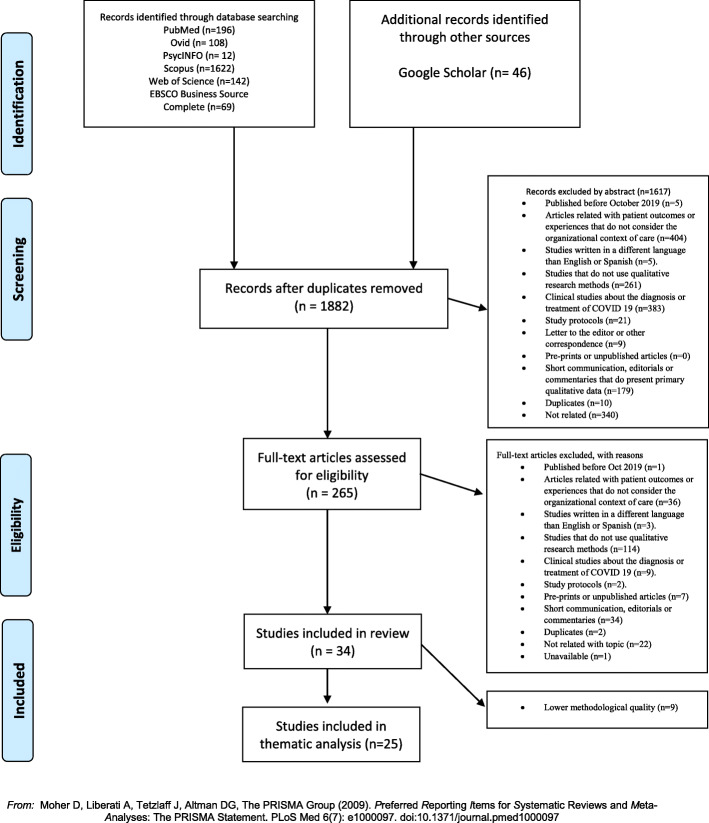
PRISMA 2009 flow diagram

The search retrieved 1882 studies after duplicates were removed. After conducting the title and abstract screening, 265 full-text articles were assessed for eligibility, 34 of which were eligible for data extraction (Fig. 1).

### Characteristics of the studies

The 34 studies were published between March and October 2020. Study characteristics are summarised in Table 1. Many of the studies related to China (*n*=6) and the USA (*n*=6) and, in general, documented experiences from Asia, Europe and South America. Different methods of qualitative data analysis were reported, but the use of thematic analysis predominated (*n*=17). Frontline healthcare workers were the primary subjects of the studies, mainly nurses and medical doctors, with fifteen studies each including these types of participants. Other healthcare workers participated, such as pharmacists, laboratory technicians, administrative workers, community health care workers and public health stakeholders. Data extracted from the qualitative studies are provided in Additional file 4.

**Table 1:** Characteristics of studies included in full-text review

	*n*= 34	%
Country		
China	6	17.65
USA	6	17.65
Italy	4	11.76
Bangladesh	2	5.88
Iran	2	5.88
Singapore	2	5.88
Spain	2	5.88
Belgium	1	2.94
Brazil	1	2.94
France	1	2.94
Israel	1	2.94
Jordan	1	2.94
Lebanon	1	2.94
Malaysia	1	2.94
Pakistan	1	2.94
UK	1	2.94
Global (cross-sectional surveys)	2	5.88
Type of study		
Qualitative, thematic analysis	17	50.00
Qualitative, case study or report	8	23.53
Mixed methods (e.g. survey with some open-ended questions)	5	14.70
Qualitative, narrative analysis	2	5.88
Qualitative, phenomenological approach	2	5.88
Participants^a^		
Nurses	15	
Physicians	15	
Pharmacists	4	
Respiratory therapists	1	
Medical assistants	2	
Physical therapist	1	
Department supervisors	3	
Laboratory technicians	3	
Social workers	2	
Administrative workers	3	
Community healthcare workers	2	
Home care agencies’ workers	2	
Public health stakeholders	2	
Unspecified	3	

^a^Adds up to more than 35 due to multiple types of participant in the studies. One cross-sectional survey involved multiple types of participant involved in delivering maternal and new born healthcare and is not included in the participant breakdown

### Thematic analysis of results by level

Applying the quality assessment criteria, nine studies were removed due to being of lower methodological quality [20–28], meaning that 25 were of sufficient quality to include in the thematic analysis. Seven of the nine studies removed were case reports. The results of the thematic analysis are divided into processes at the professional, organisational and local health system level (Table 2). The methods employed in the qualitative studies examined after the findings on responses at the three levels are described.

**Table 2:** Summary of results by theme

Professional level	Organisational level	Local system level
Front-line staff asked to adapt to COVID-19 and exhibit resilience Staff experienced shifting roles and increased workload to respond to COVID-19 Staff stress and burnout exacerbated by fear of contagion and lack of suitable protective measures, including PPE Some healthcare staff less recognised, especially home care workers Examples of innovative initiatives include telehealth and community engagement	Introduced psychological support programmes for workforce Recognition of otherwise ´unseen´ workers Emphasised open or engaging leadership Use of cross-functional teams to support care coordination Reflection on how COVID-19 relation information shared within and beyond organisation COVID-19 seen as ‘catalyst’ for overcoming typical barriers to change	System-wide training to mitigate stress Safeguarding working conditions Source of external public resources and finance, but concern about sustainability Enabled coordination of relationships across organisations Updating of regulations and contracting needed to support service-level changes (e.g. introduction of telemedicine)

#### Professional level

This level summarises the experiences and perspectives of frontline and managerial staff. Broad themes presented in primary qualitative data analysed healthcare workers’ adaptations to new roles and responsibilities, burnout and distress, “unseen” healthcare workers that were crucial to respond to COVID-19, and changes made to healthcare workers’ practice.

##### New roles and responsibilities

A frequent topic examined in the studies was changes to healthcare workers’ job scope. As multiple types of healthcare worker were required to adapt their role to support the response to COVID-19, this exposed them to unknown task environments; healthcare workers like transdisciplinary nurses perceived higher levels of stress due to the need to acquire new knowledge [29]. In the words of an interviewee, ‘when facing large-scale respiratory infectious diseases, the work regulations are completely different from daily work, which also requires an adaptation process’ [29]. In this process of adaptation, primary care practitioners perceived that they were assigned to unsuitable positions, contributing to ambiguity about their new responsibilities [30]. Changes to medical practices limited face-to-face interactions and increased administrative workload for general practitioners. In the words of some interviewees: ‘The workload is different from the usual: you need to start earlier, make a lot of phone calls, send emails in between, try to keep up to date by reading a lot, adjust your website’ [31]; ‘Corona—it is not extra work, it is completely different work’ [32].

##### Burnout, physical and psychological distress

COVID-19 presented conditions that triggered healthcare workers’ burnout and distress. For example, it was highlighted that workload increased and induced high levels of stress [33] and that this was aggravated by staff absenteeism and shortage [34]. Fear of contagion and transmission to their patients and families was a prominent concern in the narratives of many healthcare workers [32, 35–40]. This fear was a particular concern for home healthcare workers, who expressed that they posed a unique risk of spreading the disease [41]. Perceived sufficiency of personal protection equipment (PPE) mitigated burnout [33]. However, using PPE aggravated working conditions when endured for several hours [42–44]. In the words of an interviewee: ‘Wearing a mask and gloves and also using a face shield for several hours are very difficult because it cannot be tolerated for an hour, but you have to endure it in six hours and you cannot even meet your basic needs’ [35]. Participants in one study likened their front-line role to ‘preparing for war’ [32]. Self-care strategies were frequently resorted to like problem oriented coping [35] or spirituality [32, 35, 42].

##### ‘Unseen’ healthcare workers

Healthcare workers with different professions or occupations to traditional nurses or medical doctors were highlighted by some researchers as key to responding to COVID-19. Perceptions of feeling neglected relative to more visible health service staff were manifested by home care workers, as this profession was perceived as ‘a forgotten field. . . You hear people clapping, thanking doctors and nurses, even the hospital cleaning staff. . . I’m not doing this because I want praise; I love what I do. But it would be nice for people to show us gratitude’ [41]. Other participants that felt excluded from the wards due to shortage of PPE were hospital-based clinical pharmacists [45]. Less-recognised healthcare workers including nurses, hospital attendants, technicians and administrative personnel, felt that they gained ‘hyper-visibility’ during the pandemic; this ‘new hero status’ may or may not be sustained over time [46].

##### Positive changes and emergent solutions amid the crisis

The qualitative studies highlighted some positive outcomes perceived by healthcare workers. Aspects of resilience amid challenges among both hospital-based and home care staff were emphasised [42, 47]. A frequent topic was the use of technology for continuing their practices. Telehealth solutions enabled rapid communication, continuing education and raising public awareness [48]. The case of community healthcare workers manifested the challenges of developing skills to engage young people, to transfer knowledge to the citizens amid uncertainty, and adapt to changing guidelines set by national regulatory health organisations [47].

In summary, healthcare workers, particularly front-line staff, were directly affected by COVID-19 through the need to adapt to new roles or responsibilities and the experience of stress and burnout. Staff also produced creative approaches to new challenges in their roles.

#### Organisational level

This level summarises organisational responses to COVID-19 as a ‘catalyst’ for service change, supporting staff wellbeing, and adopting new approaches to leadership.

##### ‘Catalyst’ for service change

COVID-19 was regarded as a ‘catalyst’ for implementing new projects and professional teamwork [49]. In order to implement primary care video visits, traditional barriers related to provider or staff needs, health system evolution, and access and equity for patients must be overcome [49]. Other positive changes learned from COVID-19 responses were the development of communication devices with patients and families [50, 51], and fostering coordination efforts among medical leaders to deal with information ‘overload’ about COVID-19 and how to communicate it through convincing narratives [52].

##### The urge to provide psychological support

Multiple studies highlighted the urgent need to provide psychological support to healthcare workers facing the pandemic [29, 31–33, 35, 36, 38, 39, 42]. Interventions aimed at improving staff wellbeing were introduced in one hospital in Israel following the analysis of a focus group and interviews [32]. Such support extended to maintaining a focus on healthcare workers´ morale [52]; two studies in China emphasised the need to create a working environment that prioritised mutual trust, peer support and belonging among staff [42, 43]. However, one study found varying organisational support among home care agencies for their workers (e.g. information, PPE, training), exacerbating staff stress [41].

##### Changes to leadership

Adoption of an engaging or open management style during crisis was identified in several studies [35, 36, 42, 46, 52]. Hospital managers should actively acknowledge the contribution of their healthcare workers [46] and clarify the roles of individuals and teams where new protocols are established [42]. Safeguarding the working conditions of the ‘unseen’ healthcare workers was suggested, by providing paid sick time to community healthcare workers [47], and increasing their earning potential [46]. Introducing cross-functional teams for improving care coordination [30], and supporting information sharing, were suggested [52].

In summary, changes in organisational processes were oriented toward safeguarding the mental health and wellbeing of staff, although organisations varied in their commitments toward staff, potentially exacerbating staff stress. Emergent leadership processes to respond to COVID-19 included interventions to improve care coordination and information sharing within and across different organisational functions. COVID-19 was regarded a ‘catalyst’ for implementing new ways of working, including technology use.

#### Local health system level

This theme examines the roles of actors and processes at the local health system level (e.g. metropolitan area or region) which influenced the coordination of health systems’ response to COVID-19.

##### Continuous training

The emergence of new and often challenging tasks for health care professionals in responding to COVID-19 suggests a role for local system actors in supporting COVID-19-related education and training. A phenomenological study of nursing staff working in hospitals in Wuhan, China, suggested that public health training needed to be strengthened to make completing their new-found responsibilities less arduous, thereby helping to mitigate the consequences for nurses’ mental and physical health [43]. Another phenomenological study in China of doctors and nurses seconded to provide care to COVID-19 patients suggested that continuous training of front-line medical teams was needed to support their preparedness for future pandemics [42].

##### Working conditions

COVID-19 had an impact on working conditions, especially those of front-line staff delivering care. Local system actors had an important role in safeguarding working conditions. A US study of community health care workers noted that their experiences of work during COVID-19 varied with the nature of the position, the state affected, source of funding and the type of agency [47], suggesting a need for system-wide approaches to safeguard working conditions, including ‘burnout’ monitoring and management [33].

##### Providing supportive resources, but how sustainable?

The critical role of system-wide organisations, at national and local level, in providing resources, including finance, were acknowledged in enabling health systems to adapt in response to COVID-19. A ‘health system resilience’ checklist—developed in consultation with key informants in Bangladesh—underlines the perceived importance of access to internal and external emergency funding [53]. Other research identified concern among participants about the sustainability of new levels of care and services without system-wide support. A US ‘video visits’ programme in primary care coordinated by an academic medical centre was implemented rapidly with temporary federal funding, but some programme leaders raised concerns about its sustainability without such emergency funding [49].

##### Coordinating a diversity of actors

Local system actors helped to coordinate both operational relationships between, and learning activities among, a diversity of professional groups that were involved in responding to COVID-19.

A study in China suggested a need for system-wide policies to clarify the division of roles between community and primary care practitioners and support their coordination [30]. Other tasks that could benefit from system-wide coordination were developing new technology, directing financial investment, developing the capacity of the health care workforce [30] and securing access to PPE and other COVID-related medical equipment [33]. With regard to learning, an interview-based study of general practitioners in Belgium identified the importance of external sources of information on COVID-19 provided by a variety of local system organisations or groupings (including a public research institution, a professional association and social media chat groups) for updating general practitioners’ awareness and knowledge in relation to an emergent public health problem [31].

##### Reviewing and updating regulations

An important role for health system leadership was enabling the timely review and updating of regulations to allow adaptations to organisational processes and relationships in response to COVID-19. This covered activities relating to services for COVID-19 patients and other health services affected indirectly. A US study noted mixed responses among home care agencies to legal reform enabling paid leave for staff, with some raising concerns about its impact on their financial viability [34]. With regard to non-COVID-19 services, a survey and interviews with doctors in Lebanon identified some concerns about the safety and effectiveness of telemedicine, despite national ministry and professional association support, and called for regulations concerning telemedicine use to be updated [48]. While political will and resources expedited reform, adequate regulation needed to catch up.

In summary, the delivery of support for local health systems was advocated in a number of papers to enable their adaptation in response to COVID-19. Such support should be directed at the operational level of individual providers (e.g. safeguarding workforce conditions), facilitating the coordination of different health system organisations (e.g. sharing resources) and supporting information sharing and learning across professional networks and health care organisations. While checklist recommendations for improving the resilience of local health systems appear well-founded and appropriate, e.g. [53], the studies reviewed also suggest that financing and organisational challenges will need to be addressed if such recommendations for supporting resilience implemented in an ‘emergency’ context are to be sustained in the longer-term.

#### Conducting qualitative research in times of COVID-19

Undertaking and reporting qualitative research in a timely way has been challenging during the time of COVID-19, and much has been stated about this type of research lagging behind quantitative research in terms of the delivery, credibility and sharing of findings in real time [54]. Conducting fieldwork has been difficult due to pressures on health services, as well as mandatory quarantine, social distancing and other regulations worldwide affecting researchers. With regard to data collection, the majority of studies included interviews (*n*=24), undertaken face-to-face or through the use of telephone or online applications, which were often undertaken rapidly (e.g. one study [49] reported the completion of 53 interviews by a team of nine researchers within 4 days). Despite health service pressures that may preclude research opportunities, access may have been aided by involvement of practitioners, including clinical academics, who possessed links to the service organisations being evaluated (13 of the 15 authors of study [49] are qualified medical doctors). Widespread use of field research teams appears to have supported rapid data collection.

With respect to data analysis, the majority of studies (*n*=17) used some form of thematic analysis or other inductive approaches (e.g. phenomenological methods). Given the unprecedented nature of COVID-19, it is understandable that many studies relied on grounded theory approaches in order to develop context-specific insights that emerge from analysing local responses to COVID-19. That said, there was a lack of research informed by organisation theory in the studies reviewed (a notable exception was use of a crisis leadership framework [52]).

## Discussion

### Summary of findings

This is the first systematic review of primary qualitative studies to assess the experiences and perceptions of organisations and actors at multiple levels of health systems internationally in responding to COVID-19. Experiences of frontline healthcare workers internationally were the predominant perspective analysed in the articles included in this systematic review. Responses to COVID-19 have impacted on the healthcare workforce, including home care workers, through new or expanded roles, increased workload, concerns associated with contagion of COVID-19 (including availability and use of PPE), and consequent experiences of burnout and stress. Some studies reported use of self-care strategies among staff and organisational interventions to protect healthcare workers from burnout and foster their physical and mental health. Other organisational actions included a shift towards more ‘open’ leadership, improving workforce morale and trust, introducing service changes like telemedicine, and a focus on how COVID-19 related information is gathered and shared. Actions at the wider system level included training delivery, provision of resources and finance, review of healthcare workers’ conditions, and updating regulations associated with service changes and innovations. Concerns were expressed about the sustainability of changes introduced in a context of emergency, including funding, resourcing and regulation.

Additionally, this review suggests that the reporting of qualitative research could be more rigorous, especially giving more detailed explanations in the methods section. Our review excluded nine potentially relevant studies due to their being of lower methodological quality. Authors of qualitative research worldwide could benefit from following free accessible guidelines for reporting (e.g. EQUATOR Network SRQR [55] and COREQ [56] guidelines).

### Contributions to existing research

The majority of qualitative research on COVID-19 has focussed on the professional level and documented the experiences of frontline healthcare workers; narratives of exhaustion due to heavy workload, physical and emotional distress, stigma and fear of contagion are predominant themes [57]. Other systematic reviews have identified the burden on healthcare workers’ mental health from responding to pandemics and that evidence on intervention measures is limited [58, 59]. Additionally, this review highlights workforce resilience and their role in implementing innovations in ways of working and service developments. However, health systems’ reliance on, and the sustainability of, such resilience can be questioned when set against the documented impacts on health care workers’ physical and mental health.

This study complements research at the professional level by synthesising evidence on responses to COVID-19 at the organisational and local health system levels. Another systematic review has advocated the continuity of care offered by a subset of services, telehealth, in times of COVID-19 [60]. This review adds to such research on service change outcomes by characterising processes of service change in response to COVID-19 at the organisational and local system levels. Our reading of evidence from qualitative studies indicates a number of patterns of service change that appear to be particular to COVID-19. Firstly, processes led at the wider health system level, rather than at the level of individual organisations, have been key to making service adaptations in response to COVID-19. Local system level interventions include safeguarding working conditions [33, 43], facilitating inter-organisational cooperation [30] and coordinating the sharing of relevant information [31, 47]. The use of professional networks, spanning different organisations, has also supported the sharing of learning among organisations [4, 41]. Such evidence of collaboration is at odds with much of the literature on major system change, in which improvements are coordinated among multiple organisations across a population area, yet is theorised to encounter significant resistance due to the presence of multiple stakeholder interests, including recognition that structural incentives may encourage healthcare providers to prioritise their own patients’ interests over those of the wider population [61, 62]. As described above, examples of system-wide approaches to service planning and delivery in response to COVID-19 have been relatively prominent, although further evidence is needed on how individual provider organisations have engaged in such processes.

Second, service innovations have been introduced at a pace not typically associated with the healthcare sector. The introduction of innovations has been underpinned by both ‘softer’ and ‘harder’ organisational mechanisms. An example of a soft mechanism was COVID-19 as a ‘catalyst’ [49] or motivation to accelerate the introduction of service innovations (e.g. telemedicine) by overcoming barriers that typically impede change processes. Advocacy by professional associations also supported introduction of service innovation [48]. Examples of harder mechanisms to support service adaptation in response to COVID-19 included temporary funding [49], new clinical training programmes [50], system-wide information sources [31] and changes to staff contracts [34]. There is a need for alignment of soft and hard mechanisms to support the introduction and sustainment of service innovations. For instance, one study highlighted that, despite receiving advocacy from professional associations, there were concerns among some doctors that regulation of telemedicine in new service areas lagged behind [48].

Third, there has been recognition of the critical role of a variety of front-line workers that is often overlooked in change processes. In diffusion of innovations, the workforce can be regarded as a necessary obstacle to the implementation of change, with recommendations for achieving change tending to reflect rather than challenge existing power structures (e.g. prioritising engagement of powerful physicians over other types of stakeholder [62]). The qualitative studies reviewed indicate instead the burden of impact on, and significant augmentation of the roles and workload of front-line staff, including the often-invisible role of home care workers [41] and other less-recognised categories of healthcare staff [46]. Some studies highlight the consequent need for managerial interventions [30] and training [50] that supports the workforce, including mitigation of impacts on their physical and mental health [29, 32, 33, 35–37, 42–44], and improving staff morale and belonging [42, 46, 52]. Decision-making on organisational change should reflect the perspectives of those upon whom the burden of changes fall, rather than be limited to the usual stakeholders with the positional power to block or stymie change.

### Implications for policy and practice

The primary qualitative studies herein analysed suggest that emphasis should be placed on system-wide approaches to service planning and delivery. In responding to COVID-19, provider organisations in diverse settings have faced common challenges that include supporting the important role of community workers in primary care, clarifying the division of roles between community and primary care practitioners and support their coordination [30]; the development of new technology, directing financial investment and developing the capacity of the health care workforce [30]; and securing access to PPE and other COVID-related medical equipment [33]. It would be logical to address these and related challenges through a system-wide approach; public health authorities therefore need to focus on the underpinning organisational arrangements that will support horizontal and vertical coordination where this can help to address common challenges. System-wide decision-making concerning service planning and delivery should involve strong representation from the front-line workforce who have carried the heavy burden of responding to COVID-19. The implementation of rapid service innovations catalysed by COVID-19 [2], including telemedicine, will need to be reevaluated as to whether such changes in care planning, financing and delivery can or should be sustained beyond the ‘emergency’ phase.

### Review limitations

The information retrieved covered primary studies published in the period from October 2019 to October 2020 and therefore summarises qualitative evidence on early responses to the pandemic published within a year of its onset. Considering that other qualitative studies on early responses to the pandemic could take more time to write-up and be published, potentially insightful data published after October 21, 2020, are not part of the sample. Moreover, early experiences and responses to the pandemic reported in this review are likely to have evolved with time and may be captured in more recent qualitative studies. To our knowledge, this review represents the first qualitative synthesis of multi-level responses to the pandemic internationally which can help to direct ongoing qualitative research on, and policy responses to, the pandemic.

Inclusion of ‘preprint’ articles would have captured more studies undertaken within the year of COVID-19 breaking; however, due to our aim of developing insights for informing policy and practice, we excluded studies that had not already navigated peer review. There is a need to establish guidance on the use of preprint articles in systematic reviews to improve the timeliness of review findings by clarifying how judgements concerning trade-offs between the timeliness and robustness of evidence presented in such reviews are made. Primary qualitative studies were retrieved and selected from multiple countries; however, this systematic review did not identify studies reporting the experiences of actors responding to COVID-19 in two continents, Africa and Oceania.

There was a lack of research informed by organisation theory in the studies reviewed. The widespread lack of use of extant theory or frameworks makes it difficult to evaluate, at this time, the relevance of previous conceptual work on key organisational themes to the navigation of responses to COVID-19 at different levels of health systems. Relevant theoretical frameworks include theories of coordinated change, e.g. major system change [62]; how norms and values are influenced by environment “shock”, e.g. institutional change [63]; and health system resilience as professions and organisations seek to adapt to the pandemic [64]. Future qualitative studies should draw more explicitly on established theoretical frameworks that can be applied critically to the particular context of COVID-19, and then further developed in response to the empirical evidence identified, in order to better understand how health system responses to COVID-19 should be guided.

## Conclusions

COVID-19 is posing a major challenge to health systems worldwide. The majority of primary qualitative studies have focused on the experiences of frontline healthcare workers as of October 2020. These suggest a priority of safeguarding the workforce. The need for theory-informed primary research was identified at the organisational level to clarify similarities and differences between how change is conceptualised and practised before and after COVID-19. National level analyses that describe and compare responses to COVID-19 in diverse national settings are also required. Some eligible studies suffered from lower methodological quality (9/34); the reliability of case reports needs particular attention.

## Supplementary Information

**Additional file 1.** PRISMA checklist

**Additional file 2.** Search formula.

**Additional file 3.** Quality assessment for the systematic review of qualitative evidence.

**Additional file 4.** Characteristics of primary studies included in full text review and quality assessment.

## Data Availability

The datasets supporting the conclusions of this article are included within the article and its additional files.

## Abbreviations

COREQ: Consolidated criteria for reporting qualitative research
COVID-19: Coronavirus disease 2019
PPE: Personal protection equipment
SRQR: Standards for reporting qualitative research
