# Supplementary material for: Systematic review of experiences and perceptions of key actors and organisations at multiple levels within health systems internationally in responding to COVID-19
Source: Implement Sci. 2021 May 7;16:50. doi: 10.1186/s13012-021-01114-2 (PMC8103061; doi:10.1186/s13012-021-01114-2)
Supplement: Supplementary file 2 — Additional file 2. Search formula. [file 13012_2021_1114_MOESM2_ESM.docx]

**Additional file 2. Search formula.**

| **Database** | **Formula** |
| --- | --- |
| **PubMed** | Formula: (((((((coronavirus[MeSH Terms]) OR (COVID 19[Text Word])) OR (COVID-19[Text Word])) OR (COVID19[Text Word])) OR (SARS-CoV-2[Text Word])) OR (severe acute respiratory syndrome coronavirus 2[Text Word])) AND (((((((((Qualitative Research[MeSH Terms]) OR (Interview[MeSH Terms])) OR (Focus Groups[MeSH Terms])) OR (Grounded Theory[MeSH Terms])) OR (Narration[MeSH Terms])) OR (Ethnography[MeSH Terms])) OR (qualitative evaluation[Text Word])) OR (qualitative study[Text Word])) OR (qualitative[Text Word]))) AND ((((((((((((((((((((((((medical staff[MeSH Terms]) OR (healthcare provider[MeSH Terms])) OR (Community Health Workers[MeSH Terms])) OR (assessment of healthcare needs[MeSH Terms])) OR (policy making[MeSH Terms])) OR (administration and organization[MeSH Terms])) OR (stakeholders[Text Word])) OR (health systems[Text Word])) OR (healthcare organizations[Text Word])) OR (healthcare management[Text Word])) OR (healthcare professionals[Text Word])) OR (healthcare innovation[Text Word])) OR (healthcare improvement[Text Word])) OR (healthcare reform[Text Word])) OR (health system barriers[Text Word])) OR (system enablers[Text Word])) OR (system facilitators[Text Word])) OR (healthcare coordination[Text Word])) OR (Global Health[Text Word])) OR (public health administration[Text Word])) OR (agency, international[Text Word])) OR (world health organization[Text Word])) OR (international organization[Text Word])) OR (international organisation[Text Word])) |
| **SCOPUS** | Limited to 2019, 2020, 2021. Language: English or Spanish  Formula: ( ( ( TITLE-ABS-KEY ( coronavirus ) ) OR ( TITLE-ABS-KEY ( covid19 ) ) OR ( TITLE-ABS-KEY ( covid 19 ) ) OR ( TITLE-ABS-KEY ( covid-19 ) ) OR ( TITLE-ABS-KEY ( sars-cov-2 ) ) OR ( TITLE-ABS-KEY ( severe AND acute AND respiratory AND syndrome AND coronavirus 2 ) ) ) AND ( ( ALL ( qualitative AND research ) ) OR ( ALL ( interview ) ) OR ( ALL ( focus AND groups ) ) OR ( ALL ( grounded AND theory ) ) OR ( ALL ( narration ) ) OR ( ALL ( ethnography ) ) OR ( ALL ( qualitative AND evaluation ) ) OR ( ALL ( qualitative AND study ) ) OR ( TITLE-ABS-KEY ( qualitative ) ) ) ) AND ( ( TITLE-ABS-KEY ( medical AND staff ) ) OR ( TITLE-ABS-KEY ( healthcare AND provider ) ) OR ( TITLE-ABS-KEY ( community AND health AND workers ) ) OR ( TITLE-ABS-KEY ( assessment AND of AND healthcare AND needs ) ) OR ( TITLE-ABS-KEY ( policy AND making ) ) OR ( TITLE-ABS-KEY ( administration AND organization ) ) OR ( TITLE-ABS-KEY ( stakeholders ) ) OR ( TITLE-ABS-KEY ( health AND systems ) ) OR ( TITLE-ABS-KEY ( healthcare AND organizations ) ) OR ( TITLE-ABS-KEY ( healthcare AND management ) ) OR ( TITLE-ABS-KEY ( healthcare AND professionals ) ) OR ( TITLE-ABS-KEY ( healthcare AND innovation ) ) OR ( TITLE-ABS-KEY ( healthcare AND improvement ) ) OR ( TITLE-ABS-KEY ( healthcare AND reform ) ) OR ( TITLE-ABS-KEY ( health AND system AND barriers ) ) OR ( TITLE-ABS-KEY ( system AND enablers ) ) OR ( TITLE-ABS-KEY ( system AND facilitators ) ) OR ( TITLE-ABS-KEY ( healthcare AND coordination ) ) OR ( TITLE-ABS-KEY ( global AND health ) ) OR ( TITLE-ABS-KEY ( public AND health AND administration ) ) OR ( TITLE-ABS-KEY ( agency AND international ) ) OR ( TITLE-ABS-KEY ( world AND health AND organization ) ) OR ( TITLE-ABS-KEY ( international AND organization ) ) OR ( TITLE-ABS-KEY ( international AND organisation ) ) ) AND ( LIMIT-TO ( PUBYEAR , 2021 ) OR LIMIT-TO ( PUBYEAR , 2020 ) OR LIMIT-TO ( PUBYEAR , 2019 ) ) AND ( LIMIT-TO ( LANGUAGE , "English" ) OR LIMIT-TO ( LANGUAGE , "Spanish" ) ) |
| **Web of Science (MEDLINE)** | Formula: MH=Coronavirus OR TS=COVID19 OR TS=COVID-19 OR TS=COVID19 OR TS=SARS-CoV-2 OR TS=severe acute respiratory syndrome coronavirus 2 AND MH=Qualitative Research OR MH=Interview OR MH=Focus Groups OR MH=Grounded Theory OR MH=Narration OR MH=Ethnography OR TS=qualitative evaluation OR TS=qualitative study OR TS=qualitative  AND MH=medical staff OR MH=healthcare provider OR MH=Community Health Workers  OR MH=assessment of healthcare needs OR MH=policy making OR TS=stakeholders OR TS=health systems OR TS=healthcare organizations OR TS=healthcare management  OR TS=healthcare professionals OR TS=healthcare innovation OR TS=healthcare improvement OR TS=healthcare reform OR TS=health system barriers OR TS=system enablers OR TS=system facilitators OR TS=healthcare coordination OR MH=Global Health OR MH=public health administration OR MH=agency OR MH=international OR MH=world health organization OR TS=international organization OR TS=international organisation |
| **OVID-EMBASE** | Formula: not available in this database. Adapted from PubMed’s formula. |
| **EBSCO Business Source Complete** | Formula: ((medical staff OR healthcare provider OR Community Health Workers OR assessment of healthcare needs OR policy making OR ( administration and organization ) OR stakeholders OR health systems OR healthcare organizations OR healthcare management OR healthcare professionals OR healthcare innovation OR healthcare improvement OR healthcare reform OR Community Health Workers OR health system barriers OR system enablers OR system facilitators OR healthcare coordination OR Global Health OR public health administration OR agency, international OR world health organization OR international organization) OR international organisation) AND (coronavirus OR covid-19 OR covid19 OR covid OR sars-cov-2 OR severe acute respiratory syndrome coronavirus 2) AND (qualitative research OR interview OR Focus Groups OR Grounded Theory OR Narration OR ethnography OR qualitative evaluation OR qualitative study OR qualitative) |
| **APA Psycnet** | Formula:  ((MeSH: (Coronavirus)) OR (Any Field: (COVID 19)) OR (Any Field: (COVID-19)) OR (Any Field: (COVID19)) OR (Any Field: (SARS-CoV-2)) OR (Any Field: (severe acute respiratory syndrome coronavirus 2))) AND ((MeSH: (qualitative research)) OR (MeSH: (Interview)) OR (MeSH: (Focus groups)) OR (MeSH: (Grounded theory)) OR (MeSH: (Narration)) OR (MeSH: (Etnography)) OR (Any Field: (qualitative evaluation)) OR (Any Field: (qualitative study)) OR (Any Field: (qualitative))) AND ((MeSH: (medical staff)) OR (MeSH: (healthcare provider)) OR (MeSH: (Community Health Workers)) OR (MeSH: (assessment of healthcare needs)) OR (MeSH: (policy making)) OR (MeSH: (administration) AND MeSH: (organization)) OR (Any Field: (stakeholders)) OR (Any Field: (health systems)) OR (Any Field: (healthcare organizations)) OR (Any Field: (healthcare management)) OR (Any Field: (healthcare professionals)) OR (Any Field: (healthcare innovation)) OR (Any Field: (healthcare improvement)) OR (Any Field: (healthcare reform)) OR (Any Field: (health system barriers)) OR (Any Field: (system enablers)) OR (Any Field: (system facilitators)) OR (Any Field: (healthcare coordination)) OR (MeSH: (Global Health)) OR (MeSH: (public health administration)) OR (MeSH: (agency, international)) OR (MeSH: (world health organization)) OR (Any Field: (international organization))) |
| **Google Scholar** | Formula: (((((((coronavirus[MeSH Terms]) OR (COVID 19[Text Word])) OR (COVID-19[Text Word])) OR (COVID19[Text Word])) OR (SARS-CoV-2[Text Word])) OR (severe acute respiratory syndrome coronavirus 2[Text Word])) AND (((((((((Qualitative Research[MeSH Terms]) OR (Interview[MeSH Terms])) OR (Focus Groups[MeSH Terms])) OR (Grounded Theory[MeSH Terms])) OR (Narration[MeSH Terms])) OR (Ethnography[MeSH Terms])) OR (qualitative evaluation[Text Word])) OR (qualitative study[Text Word])) OR (qualitative[Text Word]))) AND ((((((((((((((((((((((((medical staff[MeSH Terms]) OR (healthcare provider[MeSH Terms])) OR (Community Health Workers[MeSH Terms])) OR (assessment of healthcare needs[MeSH Terms])) OR (policy making[MeSH Terms])) OR (administration and organization[MeSH Terms])) OR (stakeholders[Text Word])) OR (health systems[Text Word])) OR (healthcare organizations[Text Word])) OR (healthcare management[Text Word])) OR (healthcare professionals[Text Word])) OR (healthcare innovation[Text Word])) OR (healthcare improvement[Text Word])) OR (healthcare reform[Text Word])) OR (health system barriers[Text Word])) OR (system enablers[Text Word])) OR (system facilitators[Text Word])) OR (healthcare coordination[Text Word])) OR (Global Health[Text Word])) OR (public health administration[Text Word])) OR (agency, international[Text Word])) OR (world health organization[Text Word])) OR (international organization[Text Word])) OR (international organisation[Text Word])) |
