# Supplementary material for: Systematic review of experiences and perceptions of key actors and organisations at multiple levels within health systems internationally in responding to COVID-19
Source: Implement Sci. 2021 May 7;16:50. doi: 10.1186/s13012-021-01114-2 (PMC8103061; doi:10.1186/s13012-021-01114-2)
Supplement: Supplementary file 3 — Additional file 3. Quality assessment for the systematic review of qualitative evidence. [file 13012_2021_1114_MOESM3_ESM.docx]

**Additional file 3. Quality assessment for the systematic review of qualitative evidence.**

| Rating  1. Very Poor  2. Poor  3. Fair  4. Good |
| --- |

| **1** | ***Abstract and title*.** |
| --- | --- |
|  | Did they provide a clear description of the study? Good: structured abstract with full information and clear title. Fair: abstract with most of the information. Poor: inadequate abstract. Very poor: no abstract. |
| **2** | ***Introduction and aims*.** |
|  | Was there a good background section and clear statement of the aims of the research? Good: full but concise background to discussion/study containing up-to-date literature review and highlighting gaps in knowledge; clear statement of aim AND objectives including research questions. Fair: some background and literature review; research questions outlined. Poor: some background but no aim/objectives/questions OR aims/objectives but inadequate background. Very poor: no mention of aims/objectives; no background or literature review. |
| **3** | ***Method and data*.** |
|  | Is the method appropriate and clearly explained? Good: method is appropriate and described clearly (e.g. questionnaires included); clear details of the data collection and recording. Fair: method appropriate, description could be better; data described. Poor: questionable whether method is appropriate; method described inadequately; little description of data. Very poor: no mention of method AND/OR method inappropriate AND/OR no details of data. |
| **4** | ***Sampling*.** |
|  | Was the sampling strategy appropriate to address the aims? Good: details (age/gender/race/context) of who was studied and how they were recruited and why this group was targeted; the sample size was justified for the study; response rates shown and explained. Fair: sample size justified; most information given but some missing. Poor: sampling mentioned but few descriptive details. Very poor: no details of sample. |
| **5** | ***Data analysis*** |
|  | Was the description of the data analysis sufficiently rigorous? Good: clear description of how analysis was carried out; description of how themes derived/respondent validation or triangulation. Fair: descriptive discussion of analysis. Poor: minimal details about analysis. Very poor: no discussion of analysis. |
| **6** | ***Ethics and bias*** |
|  | Have ethical issues been addressed and has necessary ethical approval been gained?Has the relationship between researchers and participants been adequately considered? Good: ethics: when necessary, issues of confidentiality, sensitivity and consent were addressed; bias: researcher was reflexive and/or aware of own bias. Fair: lip service was paid to above (i.e. these issues were acknowledged). Poor: brief mention of issues. Very poor: no mention of issues. |
| **7** | ***Results*.** |
|  | Is there a clear statement of the findings? Good: findings explicit, easy to understand and in logical progression; tables, if present, are explained in text; results relate directly to aims; sufficient data are presented to support findings. Fair: findings mentioned but more explanation could be given; data presented relate directly to results. Poor: findings presented haphazardly, not explained and do not progress logically from results. Very poor: findings not mentioned or do not relate to aims. |
| **8** | ***Transferability or generalisability*.** |
|  | Are the findings of this study transferable (generalisable) to a wider population? Good: context and setting of the study are described sufficiently to allow comparison with other contexts and settings, plus high score in Q4 (sampling). Fair: some context and setting described but more needed to replicate or compare the study with others, plus fair score or higher in Q4. Poor: minimal description of context/setting. Very poor: no description of context/setting. |
| **9** | ***Implications and usefulness*** |
|  | How important are these findings to policy and practice? Good: contributes something new and/or different in terms of understanding/insight or perspective; suggests ideas for further research; suggests implications for policy and/or practice. Fair: two of the above. Poor: only one of the above. Very poor: none of the above. |

| **Classification system** |  |
| --- | --- |
| **A** | (30-36) |
| **B** | (24-29) |
| **C** | (9-23) |
