# Supplementary material for: Systematic review of experiences and perceptions of key actors and organisations at multiple levels within health systems internationally in responding to COVID-19
Source: Implement Sci. 2021 May 7;16:50. doi: 10.1186/s13012-021-01114-2 (PMC8103061; doi:10.1186/s13012-021-01114-2)
Supplement: Supplementary file 4 — Additional file 4. Characteristics of primary studies included in full text review and quality assessment. [file 13012_2021_1114_MOESM4_ESM.docx]

Additional file 4. Characteristics of primary studies included in full text review and quality assessment.

| **Reference; month of publication** | **Type of qualitative study** | **Setting (country; organisation; informants)** | **Themes** | | | **QA Score** |
| --- | --- | --- | --- | --- | --- | --- |
|  |  |  | **Professional level experiences** | **Organisational level responses** | **Local health system** |  |
| Algunmeeyn et al. 2020 (33)  September | Semi-structured face to face interviews (n=30). Thematic analysis, inductive techniques. Data collection period not specified. | Jordan; one government-owned  hospital; nurses, physicians, and pharmacists working in the emergency  department and intensive care unit COVID 19 wards. | Factors influencing healthcare workforce ´burnout´:  • Job stress among healthcare providers due to higher workload, increasing burnout.  • Staff and resource adequacy perceived by frontline healthcare providers, decreasing burnout.  • Fear of COVID-19 infection, putting their families at great risk of exposure to COVID-19, increasing burnout | • Hospitals should focus on lowering and preventing the level of burnout among their staff  • Hospitals that invest in more staff may also invest in other activities that improve quality and avoid excessive workload.  • Management should ensure presence of adequate human resources, particularly pharmacists, to spread the workload | N/A | A |
| Alizadeh et al. 2020 (35)  October | Face-to-face interviews (n=18). Inductive qualitative content analysis. Data collected from 29 February to 20 March 2020. | Iran; multiple hospitals (number unspecified) that were engaged  in treatment of patients with COVID-19; nurses and medical doctors. | • Identified series of factors influencing psychological distress:  • Occupational demands due to the nature of the disease  • Organisational demands perceived by healthcare workers as lack of efficient management  • Social demands represented in stigma and emphasis on negative news  • Personal resources such as problem-oriented coping, compassion satisfaction, spirituality,  personality traits and social support  • Social resources such as teamwork, family and friends´ support, and appreciation of nurses | • Provide interventions for timely understanding of staff mental health status  • Fulfil staff’s basic psychological needs by inspiring, strengthening, and connecting leadership  • “Engaging” management suggested as effective way of motivating, uniting, and reducing employees’ distress during the COVID-19 outbreak | N/A | A |
| Cheong et al. 2020 (45) August | One-to-one semi structured interviews (n=19). Iterative thematic analytic approach. Data collection period not specified. | Malaysia; 15 hospitals; hospital-based clinical pharmacists. | • Reassignment of clinical pharmacists´ roles were perceived as changes to their job scope  • Reduction of the time spent in the ward providing pharmaceutical care  • Other participants felt increasing workload with providing clinical pharmacy services in settings previously unknown  • The participants remained committed to providing pharmaceutical care, despite being excluded from the wards. | • Perceived role of clinical pharmacists as non-essential, support staff by the hospital administration | N/A | A |
| Daphna-Tekoah et al. 2020 (32)  September | Focus group (n=1), semi-structured in-depth interviews (n=450). Narrative analysis study using “Listening Guide methodology”. Focus group conducted on 18 March 2020. | Israel; one hospital; experts in mental health and trauma,  and staff (physicians, nurses, pharmacists, respiratory therapists, department supervisors, laboratory technicians,  social workers, and administrative workers) | • Participants compared the situation in the hospital to the experience of preparing for war  • The assignment of healthcare workers to new teams and departments whose purpose and essence were unfamiliar generated fear of contamination and uncertainties  • Hospital workers’ narratives reflected their direct exposure to traumatic events and the  pervading presence of death in the hospital  • The fear of being infected and of infecting others inside and outside the hospital, especially family  and friends, was expressed vividly  • Participants emphasised the need for physical protection and the need to fulfill basic requirements, and the need for security at the psychological and spiritual levels  • A lack of communication and information about what is happening at the hospital worried some participants  • Participants expressed a need for recognition from their direct supervisors, and the need for a managerial presence | • Hospital introduced a series of responses to COVID-19:  • Ad-hoc meetings aimed at strengthening and supporting staff in transition  • Telephone support for teams put in isolation  • Targeted short interventions for healthcare workers experiencing anxiety  • Information was made available to employees  • Opening of a 24/7 hotline for consultations and questions concerning mental or emotional distress | N/A | A |
| Fan et al. 2020 (29)  July | Mixed methods study that included semi-structured, face-to-face individual interviews (n=44) and surveys. Data collection period not specified. | China; one hospital; transdisciplinary nurses (non-respiratory or infectious disease experience previous to the pandemic) | • Awareness of nurses’ responsibilities and roles. Transdisciplinary nurses acquired new roles  • Recognition of responsibilities of transdisciplinary nursing work: acquiring new knowledge, enforcing new regulations and improving physical and psychological quality. Also, the risks for transdisciplinary nurses are even greater in the fight against the COVID-19 outbreak.  • Psychological problems caused by transdisciplinary work: these nurses have a relative lack of experience, and their psychological burdens increase remarkably under intense working pressure.  • The levels of perceived stress and perceived social supports: results of quantitative analysis showed that transdisciplinary nurses had the higher level of perceived stress, with the significantly higher perceived stress scores than non-transdisciplinary nurses, and lower level of the perceived social supports | • Pre-job training regarded as effective way for transdisciplinary nurses to adapt to epidemic prevention and control-related nursing work.  • More detailed role classification, clearer role definitions and job descriptions, and appropriate suggestions for expanded responsibilities suggested as effective methods to alleviate role ambiguity and improve work efficiency.  • Introduction of a psychological consultation platform suggested for medical workers  • Increasing the security of front-line medical workers suggested to reduce psychological pressure and maintain their mental health. | N/A | B |
| Geremia, et al. 2020 (40) September. | Face-to-face interviews (n=12). Data analysis technique: "Discourse of the Collective Subject" (DCS). Data collection period not reported. | Italy (nurses, including those with managerial roles, working in health system organisations in Santa Catarina region) | • Perceived weaknesses in technical and operational capacity of nursing staff on entering the pandemic  • Reinvention and reorganisation of teams to improve management of their units in response to COVID/19  • Nursing staff needed to update their technical knowledge in relation to the pandemic working conditions | • There is a perceived lack of leadership by health system organisations  • Lack of initiatives to improve nurses’ capacity to affront the pandemic  • Multi- and uni-professsional programmes deemed important for developing theoretical and practical knowledge  • Ambiguity over current availability of technology to support practice (e.g. access to real-time epidemiological data) | N/A | B |
| Góes et al. 2020 (36)  September | Nursing staff completed semi-structured electronic form on challenges faced in response to COVID-19. Lexicographic analysis of responses. Data collected in April 2020. | Brazil; unspecified organisations; nursing staff (n=26) within pediatric facilities | • Recurrent words used in nurses´ accounts were: patient, lack, fear, PPE, team, and assistance  • Perceived lack of training, diagnostic tests, knowledge/information concerning COVID-19, as well as the decreased number of nursing workers and lack of appreciation of the profession. | • Suggested need for managers to be more proactive in terms of promoting the wellbeing of workers  • Managerial guidelines need to be adopted for properly allocating human and material resources in the health field, including the pediatric services, in addition to providing training on standard precautions | N/A | A |
| He, K et al. 2020 (37);  April | Voluntary one-to-one and focus group interviews (n=30). Deductive approach to analysis. Data collected from 12 to 16 March 2020. | United States; 2 academic  medical centres; general surgery residents | • Residents interviewed expressed a number of concerns related to COVID-19:  • Health of their family  • Risk of transmitting COVID-19 infection to their family members  • Risk of transmitting COVID-19 infection to their patients  • Anticipated overwork for taking care of a high number of patients  • Risk of acquiring COVID-19 infection from their patients | • Increase availability of COVID-19 testing and personal protective equipment  • Shift schedule in which the minimum necessary number of residents are on clinical duty at any given time, in order to minimise their exposure in the early phases of the pandemic as well as prevent resident burnout.  • Create professional psychological support systems to respond to workers´ acute stress | N/A | B |
| He, Q et al. 2020 (43)  August | In-depth, semi-structured interviews (n=10). Phenomenological analytic approach. Data collected in February 2020. | China; unspecified hospitals; nurses from other locations which supported Wuhan for caring for  the COVID-19 patients | • Different psychological stages experienced: sense of mission and responsibility, negative feelings, team cohesion and positive energy  • Work stress and new challenges related to PPE, overloaded work and strong powerlessness.  • New concepts of caring for patients experienced as the motivation of “Being Nurses,” attentive care and companionship for infectious disease patients. | • The importance of occupational protection and timely psychological support  • The hospital should arrange the psychologist to give counseling and training on mental stress knowledge and self-adjusting techniques in time before the nurses set off to the outbreak city  • Create a new and reasonable daily schedule including designated periods to eat and rest and attend to other personal needs, and allowing social and family contact  • Administrators should be positive in guaranteeing that colleagues are caring and supportive of team members  • To develop and improve the health delivery system and hospital culture with better coordination, classification, and dynamic management with the rules of priority. | • Strengthen public health training to support physical and mental health of staff | A |
| Helou et al. 2020 (48)  July | Semi-structured interviews (n=5), and survey (n=140). Thematic analysis. Exploratory sequential mixed-methods approach. Data collection ended on 16 May 2020. | Lebanon; unspecified organisation; physicians | • Prior to the COVID-19 pandemic, Lebanese physicians were engaging in telemedicine, even though they were skeptical of its safety and effectiveness and did not believe that existing telehealth regulations were adequate.  • There was a general shift in the physicians’ perceptions regarding telehealth during the COVID-19 pandemic. Post-pandemic, physicians agree more on the need for telehealth in Lebanon  • Significant amount of skepticism and uncertainty regarding telemedicine remains. | N/A | • Most physicians remain undecided or think that existing regulations for telemedicine are inadequate  • During the COVID-19 crisis, the Lebanese Order of Physicians  and the Ministry of Public Health asked physicians to offer telemedicine services, and might have induced a feeling of safety, even with the lack of adequate regulations | A |
| Hennekam et al. 2020 (46)  September | Open-ended survey (n=164), and in-depth interviews (n=3). Inductive analytic approach. Data collected from 26 March 2020 to 17 April 2020. | France; two hospitals; nonphysician health care workers (nurses, hospital attendants, technicians, administrative personnel, social worker, physical therapist) | • Non-physician workers manifested experiences of “invisibility” before COVID-19  • Workers interpreted sudden valorisation of their activities triggered by the crisis, including the perceived temporal nature of the elevated status of their occupation turning into a “hypervisible” status  • Workers’ reactions to their newfound “hero” status included embracement, expressing ambivalence, and/or rejecting it outright. | • Suggested need to increase non-physicians´ visibility by actively acknowledging their role in the organisation, granting them opportunities for upward mobility, increasing their earning potential, encouraging employee engagement, and reducing “power distance” within and across organisational roles. | N/A | A |
| Lim and Wong, 2020 (21) May | Participatory/action research based on experience of implementing containment measures across a network of clinics.  Data collected 8 to 22 February 2020. | Singapore; private general practitioner clinics (n=50). | N/A | N/A | • All medical staff within the network were promptly updated on new developments via multiple communication channels like e-mail and secure mobile chat groups, minimising difficulties with information access.  • All medical staff were trained in the appropriate use and disposal of personal protective equipment (PPE).  • Training for health care workers was provided using one-on-one sessions and multimedia information guides.  • PPE resources were provided by both institution and government stockpiles with reassurance of adequate supplies for staff protection throughout the pandemic.  • Compliance with PPE guidelines was strictly enforced at all levels. | C |
| Rahaman et al. 2020 (22) September | Variety of data collected to understand the needs of COVID-19 testing centres, including documents, unstructured telephone interviews (15 minutes on average), and geographic information systems data. Full data collection period not specified (one interview conducted on 31 May 2020). | Bangladesh; contact persons within “several” district level offices engaged in COVID-19. | N/A | N/A | • The entire testing facility for COVID-19 is very centralised and based on district-level hospitals, which may take more than 24 h to collect the samples from rural patients and more than five days to obtain test reports.  • The testing center allocation over the country is not proportionately distributed to the population density | C |
| Liu et al. 2020 (42)  April | Semi-structured, in-depth interviews (n=13). Empirical phenomenological approach. Data collected from 10 to 15 February 2020. | China; 5 COVID-19-designated  hospitals; physicians or nurses who were  recruited from their original departments to provide  direct care and treatment for patients with COVID-19 | • Being fully responsible for patients’ wellbeing  • Challenges of working on COVID-19 wards  • Resilience amid challenges. | • Emphasise the importance of self-care, set maximal working hours and arrange shifts reasonably.  • Training, education, and improved communication are needed  • Mutual trust and respectful environments should be developed, efficient communication maintained, the role of individuals and teams clarified, organisation procedures established, and a sense of belonging fostered.  • To provide a safe working environment and sufficient protective supplies and have personnel responsible for continuous training, monitoring, and supervision of infection prevention and control  • Healthcare workers’ mental health should be continuously monitored, support systems strengthened, and professional psychological counselling and crisis interventions provided. | • Continuous medical education and training is needed to assure medical teams are adequately prepared to deal with public health emergencies | A |
| Mayfield-Johnson et al. 2020 (47)  October | Focus group (n=1)with 7 participants (n=7). Inductive techniques. Data collection period not specified. | United States; National Association of Community healthcare Workers; community health workers´ (CHW) state ambassadors | • CHW identity: changes to relationships, changes to services provided, and the impact of having a shared background with the communities they serve  • CHW resiliency in terms of creative thinking and maintaining positivity and focus  • Consequences of COVID-19 like sudden changes, personal challenges and sacrifices, and issues related to the political environment that hindered building trust in the communities.  • Technology challenges like developing skills, and the connections to younger generations.  • Resources and support including public health information, community resources, fellow CHWs, social support, and financial support.  • Stressors and self-care in response  • Unexpected positive outcomes of COVID-19 including using technology to support their work, gaining access to new resources, development of skills, and personal change. | • Some participants received paid sick time for quarantining, and mental health service  • CHWs are considered essential employees and have remained employed throughout the entire pandemic. | • Employment varied with the nature of the position, the state affected, source of funding, and the type of agency  • Support included webinars and online meetings to keep CHWs informed, safe spaces to debrief and discuss issues important to CHWs, platforms for discussing issues related to CHW experiences and capabilities and keeping CHW issues in the forefront with other health and human services professionals. | A |
| Meyer et al. 2020 (53) August | Scoping review of the literature, semi- structured interviews (n=19) and a workshop. Deductive analysis. Dates of interviews not specified. Checklist workshop conducted 20-21 March 2019. | Bangladesh (workshop); public health stakeholders from 16 different organisations | N/A | N/A | • Checklist for health system resilience to infectious disease outbreaks and natural hazards  • Core health system capacities and capabilities  • Critical infrastructure and transportation  • Financing  • Barriers to accessing health services  • Communication, collaboration, coordination and partnerships  • Leadership and command structure  • Surge capacity  • Risk communication  • Workforce  • Infection control. | A |
| Mohammadi et al. 2020 (51)  July | Semi-structured interviews via video call with doctors and nurses (n=17). Content analysis of interview data. Data collected February to April 2020. | Iran; single site; doctors and nurses providing care on infection unit for COVID-19 patients | • Front-line staff focused on respect for hospitalised patients´ dignity (including psychological privacy, avoiding stigmatisation and pitying behaviors, and respect for patients’ preferences) | • Comprehensive support package provided to patients, including family, medical, social, and spiritual aspects.  • Provide ´peaceful´ clinical environment to patients that is clean, quiet, and psychologically safe | N/A | A |
| Rowe et al., 2020 (34)  July | Open-ended electronic survey of home care agencies (HCAs) (n= 1204). Inductive analysis of responses using constant comparative techniques. Survey completed in March 2020. | United States; survey sent to membership of a national HCA association, with responses received from HCAs in 46 states. | • Increased staff absenteeism due to illness, childcare responsibilities, fear about COVID-19, and confusion about ´stay-at-home´ policies. | • Challenges recruiting new or replacement caregivers  • Lacking supplies for responding to COVID-19 (e.g. PPE, disinfectant)  • Decreasing cash flow and increasing costs (e.g. paying for sick leave)  • Diversity of protocols established for surveillance of symptoms and infection control | • Paid Time Off (PTO) or sick leave mandated by law in some states.  • HCAs felt that some of their caregiver employees were dedicated to patients and would not abuse PTO policies.  • Other HCA reported concerns over PTO and its financial impact on the organisation. | A |
| Salas-Vallina et al. 2020 (23) August | Semi-structured interviews (n= 42). Inductive analysis. Data collection period not specified. | Spain; heads of medical units and nurses attending Covid-19 patients. Sampled services where contagion rates were particularly high or low. | shared leadership among team members plays an important role in low COVID-19 contagion rates among health  professionals.  shared leadership among team members plays an important role in low COVID-19 contagion rates among health  professionals.  shared leadership among team members plays an important role in low COVID-19 contagion rates among health  professionals.  • Physician trust, shared understanding, involvement, and team input matter, understood as aspects of “shared leadership” within medical units. | shared leadership among team members plays an important role in low COVID-19 contagion rates among health  professionals.  • Heads of low contagion units reportedly “promoted participative decision making, trust, and autonomy” (p.1608).  • High contagion unts reportedly showed “frequent conflict situations, revealing a lack of trust, and a lack of willingness to cooperate and provide support to each other” (p.1608). | N/A | C |
| Amin (2020) (20) May | Multiple-choice questionnaires (n=250). Data collection period not specified. | Pakistan; healthcare professionals who are directly treating quarantined patients at different hospitals. | Confinement within the home or between work and home, not being able to see friends, not being able to shop for basic necessities of everyday life enhanced their feeling of distance from the outside world.  • Confinement within the home or between work and home, not being able to see friends, not being able to shop for basic necessities of everyday life enhanced their feeling of distance from the outside world.  • About more than half healthcare professionals do not have the right information about infection control measurements | • A comprehensive psychological trauma management  program for understanding of psychological effects of traumatic situations to healthcare staff and via  telephone psychiatric assistance to address their psychological issues with the specialist mental health team. | N/A | C |
| Shalhub et al. 2020 (38) June | Cross-sectional survey of vascular surgeons on stress and coping strategies. Survey also included qualitative component via using open-ended questions.  Data collected 14 April to 24 April 2020. | 58 countries; majority of respondents from from US, Brazil, and Mexico; 1609 responses from vascular surgeons. Qualitative data: 370 free text responses from 229 respondents. | • Drastic reduction of personal revenue and concern about financial viability of their practice and institutions.  • Worry and concern for others (e.g. reluctance of patients to present to the hospital because of fear; pandemic’s impact on trainees)  • Stress associated with uncertainty concerning the disease process, the future practice of medicine, and lack of clear guidance.  · Experience significant burnout. | N/AN  N/A | N/A | B |
| Semaan et al. 2020 (39) June | Cross-sectional study. Data collected using questionnaire. Qualitative thematic analysis of free-text responses. Data collected 24 March and 10 April 2020. | Respondents from 81 countries; 725 responses from health professionals providing maternal and newborn health care. | • Respondents in high-income countries more commonly reported available/updated guidelines, access to COVID-19 testing and dedicated isolation rooms for confirmed/suspected COVID-19 maternity patients.  • Levels of stress increased among health professionals globally, due to changed working hours, difficulties in reaching health facilities and staff shortages.  • Respondents worried about the impact of rapidly changing care practices on health outcomes (e.g. reduced access to antenatal care, fewer outpatient visits, shorter length of stay in facilities after birth, banning birth companions, separating newborns from COVID-19 positive mothers and postponing routine immunisations). | Most facilities (62%) reportedly designated a COVID-19 liaison person/teamN/A  N/A | N/A | B |
| Srinivasan et al. 2020 (49)  October | Semi-structured interviews (n=53). Data collected from 23-26 March 2020.  Thematic analysis using a grounded theory approach. | United States; interviews with medical assistants, nurses, medical providers, technologists and administrators, across six primary care sites participating in a newly established ´video visits´ programme within an Academic Medical Centre. | • COVID-19 regarded as 'catalyst' for implementing new projects and professional teamwork  • Introduction of video visits' widely welcomed for overcoming patient safety issues, but some concerns - largely linked to speed of introducing new system - about missing patient contact, training, efficacy of decision-making, new roles, and sustainability.  • Potential inequality among staff types emerging as providers could work from home, while MAs could not | • Leadership prioritised COVID-19 related projects ('competing priorities' for support experienced previously) | • Temporary federal funding aided implementation (concerns from interviewees about whether this will be sustained) | A |
| Sterling et al. 2020 (41) August | Semi-structured interviews with home care workers (n=33). Data collected from March to April 2020. Data analysis followed grounded theory approach. | United States; workers within home care agencies in New York city. | • Considered 'essential, yet 'invisible' workers that continued to care for patients, while also taking on new routines to control spread of COVID-19  • Concern about transmission of COVID-19 to their patients and contracting the virus themselves.  • Staff faced dilemmas concerning providing care to 'risky' cases while being aware of patients' needs | • Varying levels of support from employing agencies reported (e.g. information, PPE, training), exacerbating staff stress.  • In response to perceived lack of organisational support, staff sought alternative sources of support, including news/social media, peer support, and securing their own PPE | N/A | A |
| Tanzi et al., 2020 (50) July | Single organisational case study drawing on audio recordings of team meetings and field notes. Data collected March 2020. | Italy: palliative care and infectious disease units of a hospital; participants (31) were physicians and nurses on both units | • Staff identified emergent solutions to challenges in patient care presented by COVID-19, including communication devices, tailoring the ´overload´ of information to patients, shared vulnerability with patients, and caring for families of patients. | • Value of delivering lectures and other forms of training (e.g. peer feedback) on emerging topics including new aspects of clinical care, including interaction between hospital physicians and primary care specialists. | N/A | A |
| Tham et al. 2020 (52) June | Semi-structured interviews (n=24). Grounded theory using constant comparative method. Data collected from 2 to 20 March 2020. | Singapore; physicians with leadership roles in a range of specialisms in a single hospital. | • Physician leaders navigated ´crisis leadership´ through sensemaking (of information overload), decision-making (maintaining the ´big picture´ in operational changes), and meaning-making (´take charge of the narrative´). | • Leaders encouraged cross-functional teams to overcome horizontal and vertical boundaries (instant messaging support communication among emergent teams)  • Collective ´sensemaking´ of information needed that involves top-management and middle managers  • Communication within and beyond the hospital was proactively and carefully managed  • Recognition of downsides of oversharing information by managers  • Awareness of need to maintain staff morale | N/A | A |
| Torri et al. 2020 (24) May | “Case report” based on data collected through two online meetings in March and April 2020 involving at least 25 public health professionals. SWOT analysis of response strategies and operations derived from themes identified by the authors. | Italy (Italian Public health response to COVID-19) | • Rapid change, perceived inconsistency, and bureaucratic rigmarole (especially initially) in technical orders and clinical protocols. | N/A | • National influenza surveillance network that is supported by regional reference laboratories.  • Advice and support for risk assessment and health surveillance in workplaces and other community settings through all Italy.  • Joint integrated response with primary care teams for detection, assessment, rapid reporting, and active surveillance of suspected or confirmed COVID-19 cases. | C |
| Verhoeven et al. 2020 (31) June | Semi-structured interviews (n=132). Thematic analysis using framework approach. Data collected from 24 to 31 March 2020. | Belgium; 132 interviews with general practitioners (GPs) in primary care within Flanders. | • GPs reported adjustments to how they managed their role and patient care (primary contact with patients is by telephone, increased administrative workload, more collaboration with psychologists and psychiatrists in primary care) | • Difficulties were reported with dealing with chronic conditions and organising acute psychological care  • Impact of the decrease in physical consultations on GPs’ income | • External sources of information for healthcare providers are Sciensano (a public research institution dedicated to science and health), Domus Medica (the Flemish organisation of GPs), and also informal chat groups on social media | A |
| Wexner et al. (2020) (27) July | Expert interviews (n=3) conducted on 31 March 2020. | Spain and Italy, prominent surgeons reporting on experiences within their own hosptials | • Some health care workers had fallen ill with COVID-19, one estimate suggested 13% across Spain were infected at the time of the interview. | • Crisis Unit established in one hospital that meets daily to make adaptive change within the organisation (e.g. used simulation centre to train staff)  • Shifts were rearranged in Italy because PPE was too heavy to carry around for eight hours.  • Hotels hired for health care professionals who do not want to return home and potentially expose their families to COVID-19 (Spain)  • Using social media to distribute reliable information on COVID-19 to staff and patients (Spain) | N/A | C |
| Wexner et al. (2020) (25) September | Expert interview (n=1) conducted on 21 April 2020. | United States, head of the American College of Surgeons | N/A | • Teleconferencing used to convene daily “incident command” meetings, participation up. | •The College mainly used their existing infrastructure to respond to the pandemic, and collaborated with all disciplines of medicine that needed to involved in patient care.  • The College coordinated the development of triage guidelines to “ramp down” surgery while in the pandemic. | C |
| Wexner et al. (2020) (26) July | Expert interviews (n=2) conducted on 2 and 9 April 2020. | United Kingdom, prominent surgeons | • Surgeons should always wear full protective equipment during any surgical procedure. | • Repurposing and retraining within hospitals to increase critical care support  • Where surgeons are redeployed, employing organisations should seek permission and provide full training (e.g. how to operate ventilators)  • Majority of elective and routine work has stopped | • Royal colleges agreed on standard operating cases for prioritising COVID/19 cases  • Campaigning to acquire additional surgical capacity through return of retired doctors and early graduation of medical students  • Surgeons have been consulting colleges for advice related to “wellbeing”; helpline and training courses available  • Existing geographical networks of hospitals coordinating changes to cancer services | C |
| Xu et al. (2020) (30) August | Semi-structured interviews (n=21). Thematic content analysis. Data collected from 12 February to 10 March 2020. | China; primary care practitioners in community health centres or townships in four provinces. | • Primary care practitioners took on new responsibilities (screening suspected cases, visiting residents in quarantine, contact tracing and monitoring, and surveillance at checkpoints)  • Some practitioners felt overburdened and assigned to unsuitable positions, confusion, limited effective scheduling, excessive inspection and meetings.  • Interviewees reported difficult tasks and inadequate capacities (like more hours working) and inexperienced community workers and insufficient cooperation.  • Psychological impacts on primary care practitioners included preoccupation with sacrifices of the health system, fatigue, and concern about risk of infection. | • Primary care practitioners worked in collaboration with community workers and community police in a “Joint Defense Team”, led by the neighborhood committee.  • Recommends improving management and supervision to avoid long working hours and confusion in the primary health practitioners    • Recommends optimising the workflow, so paperwork and reporting do not slow the process of epidemic control. | • Community workers and community police provide nonmedical support to residents in quarantine.  • Recommends facilitating cooperation among community workers to help identify the division of responsibilities.    • Recommends strengthening the health care system like developing new technology, more financial investment and improving capacity of healthcare personnel | A |
| Yan and Zhao (2020) (28) July | Collection of documentary evidence, including publicly available data, websites, and guidelines provided by “crisis management” practitioners. Data collected from 30 December 2019 to 24 January 2020. Analytical framework based on the theory of “policy participation”. | China; “crisis management” practitioners in Wuhan. | N/A | N/A | • Three main stages were identified according to the containment situation. Stage 1. characterised by limited knowledge of the transmission dynamics of the virus and a consequently weak response, the health department assumed the virus was pneumonia and reported cases of this. Only isolation measurements recommended. Stage 2. the disease spreads rapidly, the health department didn't notify new cases. During stages one and two, it was said that there were insufficient early warnings that led to more sick people. Stage 3 containment efforts were intensified, with pneumonia cases growing in China and neighbouring countries. Wuhan placed under heavy lockdown; donations of medical equipment made to the health care system; purpose built hospital.    • Medicine organised adjunct treatments involving traditional herbal therapy. The Ministry of Science and Technology launched a specialised fund aimed at encouraging the development of medicines and a vaccine for COVID-19. | C |
| Zhang et al. (2020) (44)  June | Semi-structured interviews (n=23). Inductive data analysis technique. Data collected 9 February to 15 March 2020. | China; nurses in a single hospital in Wuhan. | • Psychological impact on nurses passed through three linear stages (ambivalence, emotional exhaustion, and renewal).  • Topic of PPE caused physical distress, aggravating nurses´ psychological stress (inc. fear of infection) | • Support from nursing managers was reported to alleviate stress (e.g. protection training, work shift adjustment, psychological comfort, and two-way communication) | N/A | A |

Note: References in greyed out rows (20-28) were judged to be of lower methodological quality and were not included in the thematic analysis.
